# Metabotropic GABA signalling modulates longevity in *C. elegans*

**DOI:** 10.1038/ncomms9828

**Published:** 2015-11-05

**Authors:** Lei Chun, Jianke Gong, Fengling Yuan, Bi Zhang, Hongkang Liu, Tianlin Zheng, Teng Yu, X. Z. Shawn Xu, Jianfeng Liu

**Affiliations:** 1 College of Life Science and Technology, Collaborative Innovation Center for Brain Science, Huazhong University of Science and Technology, Wuhan, Hubei 430074, China; 2 Department of Molecular and Integrative Physiology, Life Sciences Institute, University of Michigan, Ann Arbor, Michigan 48109, USA

## Abstract

The nervous system plays an important but poorly understood role in modulating longevity. GABA, a prominent inhibitory neurotransmitter, is best known to regulate nervous system function and behaviour in diverse organisms. Whether GABA signalling affects aging, however, has not been explored. Here we examined mutants lacking each of the major neurotransmitters in *C. elegans*, and find that deficiency in GABA signalling extends lifespan. This pro-longevity effect is mediated by the metabotropic GABA_B_ receptor GBB-1, but not ionotropic GABA_A_ receptors. GBB-1 regulates lifespan through G protein-PLCβ signalling, which transmits longevity signals to the transcription factor DAF-16/FOXO, a key regulator of lifespan. Mammalian GABA_B_ receptors can functionally substitute for GBB-1 in lifespan control in *C. elegans*. Our results uncover a new role of GABA signalling in lifespan regulation in *C. elegans*, raising the possibility that a similar process may occur in other organisms.

## Abstract (web summary)

The *C. elegans* nervous system influences organismal lifespan but mechanistic details are poorly understood. Here, Chun *et al*. show that the neurotransmitter GABA regulates worm lifespan by acting on GABA_B_ receptors in motor neurons, which activate the transcription factor DAF-16 in the intestine.

## Graphical abstract

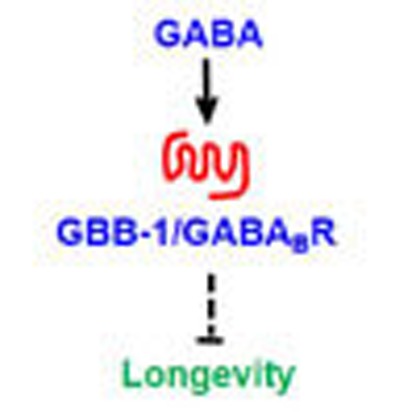

Aging is a complex physiological process modulated by a multitude of genetic and environmental factors^1^,^2^. Recent work has revealed an important role of the nervous system in modulating aging^1^,^3^,^4^. For example, in insulin/IGF-1 signalling, one of the best characterized longevity-regulatory pathways, the DAF-2 insulin/IGF-1 receptor primarily acts in the nervous system to regulate lifespan in *Caenorhabditis elegans*^5^. Interestingly, work in mice revealed a similar mechanism^6^. Environmental factors also affect aging by impinging on the nervous system^1^,^3^,^4^,^7^,^8^. Apparently, neurons can act cell nonautonomously to modulate aging, and neuroendocrine signalling plays an important role in this process^1^,^3^,^4^,^7^,^8^. However, whether and how neurotransmitter signalling modulates aging is poorly understood.

GABA is the primary inhibitory neurotransmitter in the mammalian brain^9^,^10^. It is critical for the function and development of the nervous system and plays a key role in behavioural control^11^,^12^,^13^,^14^. GABA acts through both ionotropic and metabotropic receptors^15^,^16^. As ion channels, ionotropic GABA_A_ receptors mediate the acute, fast actions of GABA^17^,^18^. By contrast, metabotropic GABA_B_ receptors are G protein coupled receptors (GPCRs) that execute the slow, long-lasting effects of GABA^19^,^20^,^21^,^22^.

*C. elegans* is a popular model organism widely utilized to study the biology of aging, featuring a short generation time and lifespan, as well as conserved genetic mechanisms that regulate longevity^1^,^2^. *C. elegans* has also been widely used as a genetic model for neurobiology^23^. Although *C. elegans* possesses a relatively small nervous system, it produces all the major neurotransmitters (for example, ACh, glutamate, GABA and biogenic amines) and their cognate receptors, all of which are encoded by evolutionarily conserved gene families^23^. While the role of neurotransmitters in controlling behaviour and nervous system development and function has been extensively characterized, little is known about their role in aging.

Here we interrogated the role of neurotransmitters in lifespan control in *C. elegans* and found that GABA regulates lifespan through GABA_B_ receptor signalling. We further identified a genetic pathway that cell nonautonomously transmits longevity signals from the GABA_B_ receptor in motor neurons to the transcription factor FOXO/DAF-16 in the intestine. Our results identify a novel function of GABA beyond its conventional role in modulating animal behaviour and nervous system function and development.

## Results

### Deficiency in GABA signalling extends lifespan

**Figure 1:**
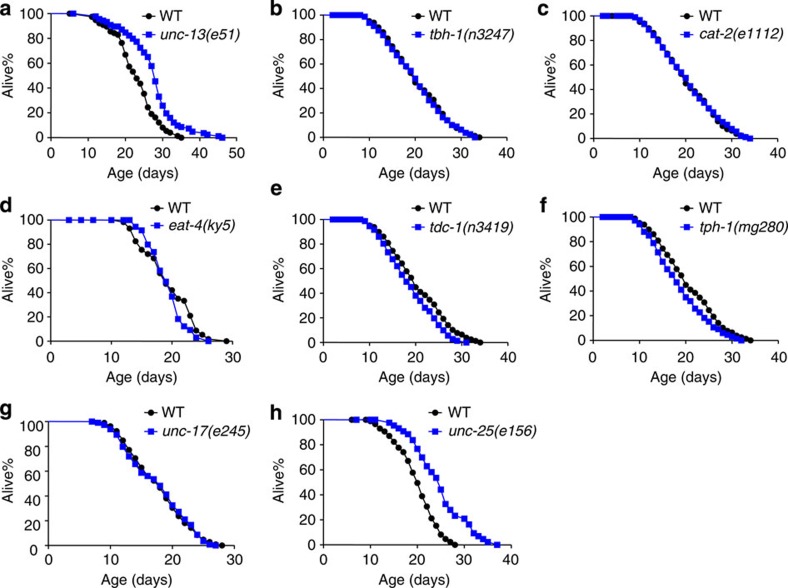
Loss of GABA signalling promotes lifespan. (**a**) *unc-13* mutant worms are long-lived. UNC-13 is required for the release of neurotransmitters (log-rank test, *P*<0.001, *n*=74–82 for different genotypes). (**b**–**g**) Loss of octopamine (b), dopamine (c), glutamate (d), tyramine (e), serotonin (f) or acetylcholine signalling (g) modestly affect lifespan (log-rank test, *P*=0.755, *P*=0.811, *P*=0.371, *P*=0.046, *P*=0.069 and *P*=0.912, respectively. *n*=44–114 for different genotypes). (**h**) Loss of GABA signalling extends lifespan (log-rank test, *P*<0.001, *n*=57–85 for different genotypes). All lifespan assays were carried out at 20 °C and were repeated at least twice. 5-Fluoro-2′-deoxyuridine (FUDR) was included in assays involving *unc-13* and *unc-17* mutant worms, which are defective in egg-laying. Please see Supplementary Table 1 for detailed statistical analysis of lifespan data.

The nervous system of *C. elegans* hermaphrodites is relatively simple^23^,^24^, making it convenient to characterize the role of neurotransmission in lifespan control. We first assayed the lifespan of *unc-13* mutant worms that are defective in synaptic transmission^25^,^26^. These mutant animals are long lived (Fig. 1a and Supplementary Table 1), consistent with previous results^27^. The fact that *unc-13* mutant animals are long-lived indicates that deficiency in neurotransmitter signalling extends lifespan. To ascertain which neurotransmitter(s) may contribute to this effect, we examined mutant animals defective in each of the major neurotransmitters, including *unc-17* (ACh), *eat-4* (glutamate), *cat-2* (dopamine), *tph-1* (serotonin), *tdc-1* (tyramine), *tbh-1* (octopamine) and *unc-25* (GABA)^28^,^29^,^30^,^31^,^32^,^33^ (Fig. 1b–h). Among these mutants, *unc-25* worms lived the longest life, showing the most pronounced phenotype (Fig. 1h), while others only had modest or no effect (Fig. 1b–g). While these data by no means exclude a role of other neurotransmitters, it reveals an important function of GABA signalling in lifespan control.

### Loss of the GABA_B_ receptor gene *gbb-1* extends lifespan

**Figure 2:**
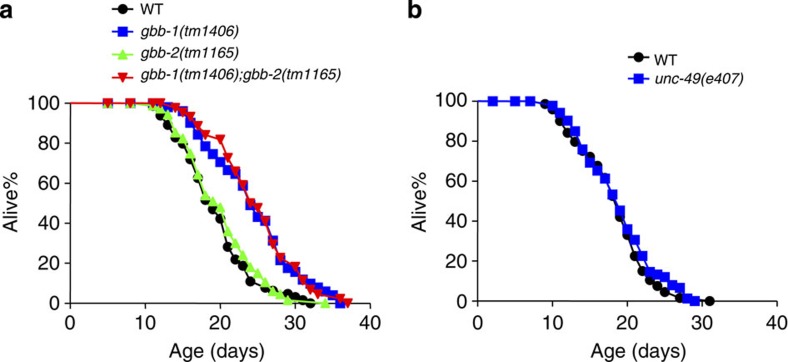
Loss of the GABA_B_ receptor gene *gbb-1* extends lifespan. (**a**) *gbb-1* mutant worms are long-lived, but *gbb-2* mutant worms are not (log-rank test, *P*<0.001, *gbb-1* mutant worms compared with wild type; *P*=0.518, *gbb-2* mutant worms compared with wild type; *P*<0.001, *gbb-1;gbb-2* mutant worms compared with wild type. *n*=44–67 for different genotypes). (**b**) *unc-49* mutant worms show normal lifespan (log-rank test, *P*=0.353, *n*=67–76 for different genotypes). All lifespan assays were carried out at 20 °C and were repeated at least three times. Please see Supplementary Table 1 for detailed statistical analysis of lifespan data.

GABA acts through both ionotropic and metabotropic receptors. The *C. elegans* genome encodes two metabotropic GABA_B_ receptor genes, *gbb-1* and *gbb-2*, which are highly homologous to their mammalian counterparts^34^. We found that *gbb-1* but not *gbb-2* mutants were long-lived (Fig. 2a). *gbb-1;gbb-2* double mutant was indistinguishable from *gbb-1* single mutant (Fig. 2a), indicating a specific role of *gbb-1* in regulating lifespan. By contrast, mutant worms lacking *unc-49*, which encodes the sole evolutionarily conserved GABA_A_ receptor family member^35^, were normally lived (Fig. 2b). These results identify a role for the GABA_B_ receptor gene *gbb-1* in lifespan control.

**Figure 3:**
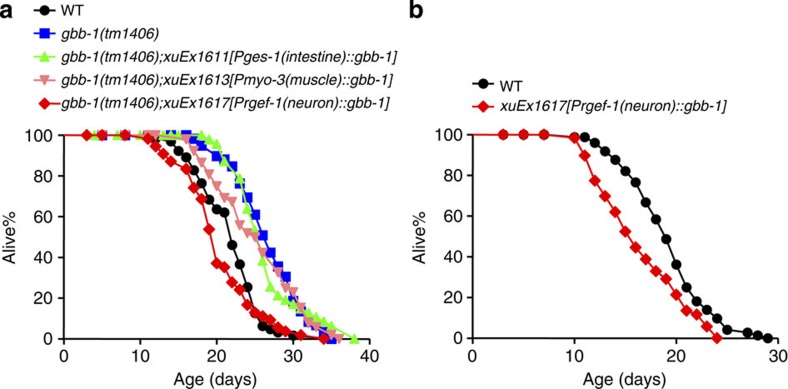
*gbb-1* acts in neurons to regulate lifespan. (**a**) Transgenic expression of *gbb-1* in neurons (log-rank test, *P*=0.198), but not in the intestine (log-rank test, *P*<0.001) or muscles (log-rank test, *P*<0.001), suppresses the long-lived phenotype of *gbb-1* mutant worms (log-rank test, *P*<0.001). The *rgef-1*, *ges-1* and *myo-3* promoters were used to drive *gbb-1* cDNA expression in neurons, intestine and muscles, respectively^61^,^62^,^63^ (*n*=47–63 for different genotypes). (**b**) Overexpression of *gbb-1* cDNA in neurons shortens lifespan (log-rank test, *P*=0.001, *n*=53–72 for different genotypes). Each plasmid DNA listed above was injected at a concentration of 50 ng μl^−1^. All lifespan assays were carried out at 20 °C and were repeated at least three times. Please see Supplementary Table 1 for detailed statistical analysis of lifespan data.

*gbb-1* is enriched in the nervous system but is also found to be expressed in other tissues such as muscle and intestine^34^. We therefore asked where *gbb-1* acts to regulate lifespan. Using tissue-specific promoters, we found that expression of *gbb-1* cDNA in neurons, but not in muscle or intestine, rescued *gbb-1* lifespan phenotype (Fig. 3a). Thus, *gbb-1* appears to act in neurons to regulate lifespan.

We crossed the *gbb-1* neuronal transgene into wild-type background and found that it rendered worms short-lived (Fig. 3b). Thus, overexpression of *gbb-1* shortens lifespan. This is consistent with our mutant data that loss of *gbb-1* extends lifespan, providing further evidence supporting that *gbb-1* regulates longevity.

### GBB-1-dependent lifespan regulation requires DAF-16/FOXO

**Figure 4:**
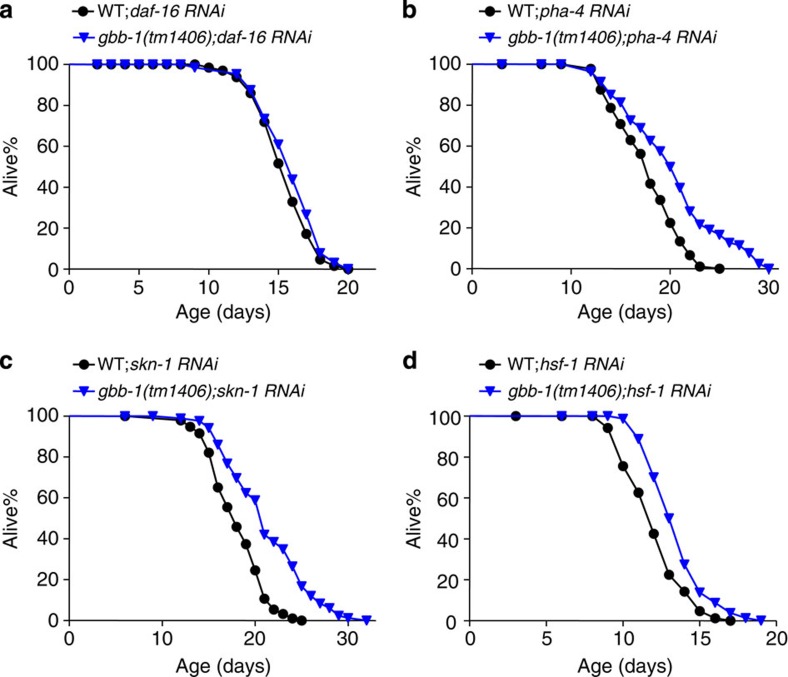
GBB-1-dependent lifespan regulation requires the FOXO transcription factor DAF-16. (**a**) *daf-16* RNAi fully suppresses the long-live phenotype of *gbb-1* mutant worms (log-rank test, *P*=0.502, *n*=78–91 for different genotypes). (**b**–**d**) RNAi of *pha-4* (**b**, log-rank test, *P*<0.001), *skn-1* (**c**, log-rank test, *P*<0.001) or *hsf-1*(**d**, log-rank test, *P*<0.001) fail to suppress the long-lived phenotype of *gbb-1* mutant worms (*n*=79–94 for different genotypes). For RNAi experiments, NGM plates included carbenicillin (25 μg ml^−1^) and IPTG (1 mM). HT115 bacteria-carrying vector or RNAi plasmid were seeded on RNAi plates 2 days before the experiment. Worms were fed RNAi bacteria from the egg stage. All RNAi lifespan assays were carried out at 20 °C, and were repeated at least three times. Please see Supplementary Table 1 for detailed statistical analysis of lifespan data.

We wondered how *gbb-1* controls lifespan. Various genetic and environmental factors regulate lifespan by converging on a subset of transcription factors^1^. We therefore examined the major transcription factors known to regulate lifespan, and found that RNAi of *daf-16* completely suppressed the lifespan-extending phenotype of *gbb-1* mutant worms (Fig. 4a), while RNAi of other transcription factors such as *hsf-1, skn-1* and *pha-4* did not (Fig. 4b–d). Thus, *daf-16* is required for the function of *gbb-1*, suggesting that *gbb-1* signals to *daf-16* to regulate lifespan.

### Gi/o-PLCβ transmits longevity signals from GBB-1 to DAF-16

DAF-16 is best known to act downstream of insulin/IGF-1 signalling to regulate lifespan. Does GBB-1 genetically interact with insulin/IGF-1 signalling? To test this, we assayed the lifespan of *gbb-1;daf-2* double mutant. *daf-2* encodes the sole worm insulin/IGF-1 receptor homologue^36^. The double mutant exhibited a lifespan significantly longer than both *gbb-1* and *daf-2* single mutants (Supplementary Fig. 1), suggesting that *gbb-1* and *daf-2* may act in different pathways. However, as a reduction-of-function allele of *daf-2* was used here (*daf-2* nulls are lethal), we did not further test this model. Thus, we do not rule out the possibility that *gbb-1* and *daf-2* may act in the same or overlapping pathways.

**Figure 5:**
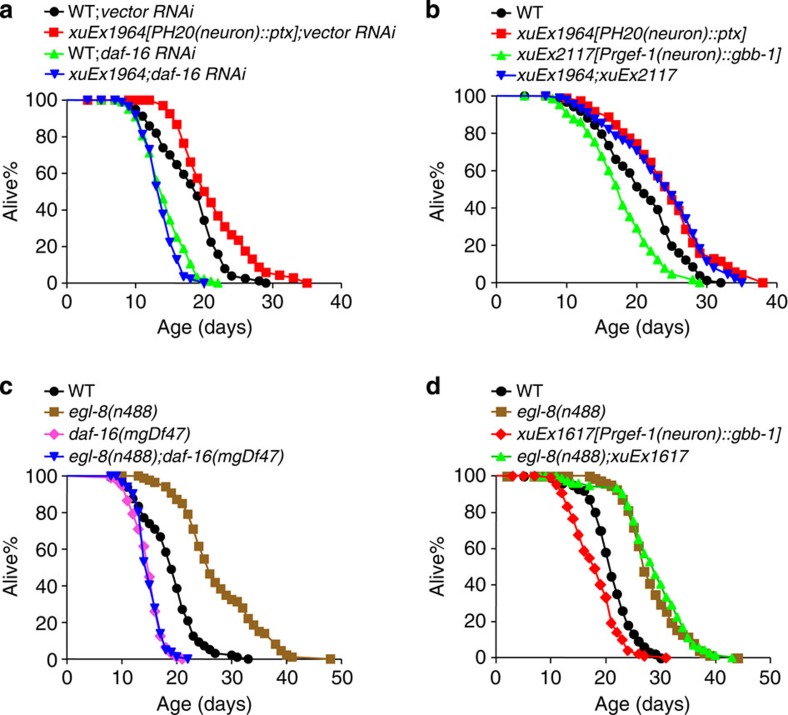
G protein-PLCβ signalling transmits longevity signals from GBB-1 to DAF-16. (**a**) Inhibition G protein function by transgenic expression of PTX in neurons extends lifespan (log-rank test, *P*<0.001), which can be fully suppressed by *daf-16* RNAi (log-rank test, *P*=0.046). The H20 promoter drives expression of PTX in neurons^64^ (*n*=68–96 for different genotypes). (**b**) Transgenic expression of PTX fully suppresses the short-lived phenotype caused by *gbb-1* transgene (log-rank test, *P*<0.001, *xuEx2117* compared with the wild type; *P*=0.56, *xuEx1964;xuEx2117* compared with *xuEx1964*. *n*=65–88 for different genotypes). (**c**) Loss of *egl-8* extends lifespan, which can by fully suppressed by *daf-16(mgDf47)* mutation (log-rank test, *P*<0.001, *egl-8* mutant worms compared with the wild type; *P*=0.781, *egl-8;daf-16* mutant worms compared with *daf-16* mutant worms. *n*=57–115 for different genotypes). (**d**) Loss of *egl-8* fully suppresses the short-lived phenotype caused by *gbb-1* overexpression (log-rank test, *P*<0.001, xuEx1967 compared with the wild type; *P*=0.382, *egl-8;xuEx1967* compared with *egl-8* mutant worms. *n*=72–102 for different genotypes). All lifespan assays were carried out at 20 °C and were repeated at least twice. 5-Fluoro-2′-deoxyuridine (FUDR) was included in assays involving *egl-8* mutant worms, which show a defect in egg laying. Please see Supplementary Table 1 for detailed statistical analysis of lifespan data.

Then how does GBB-1 signal to DAF-16? As a GPCR, GBB-1 is unlikely to communicate with DAF-16 directly. We then sought to identify genes that transduce longevity signals from GBB-1 to DAF-16. As the GABA_B_ receptor is known to be coupled to Gi/o-PLCβ signalling^10^,^37^, we examined this pathway. The worm genome encodes one Go orthologue, *goa-1* (refs 38, 39), and at least half a dozen Gi-related genes^40^. However, unlike *gbb-1*, *goa-1* mutant worms are not long-lived (Supplementary Fig. 2), suggesting the presence of functional redundancy from other Gi/o genes. To overcome this difficulty, we attempted to block Gi/o signalling using PTX (Pertussis toxin) by expressing it as a transgene in worm neurons. We found that inactivation of Gi/o by PTX extended lifespan (Fig. 5a). This PTX-dependent lifespan extension also required *daf-16*, as it can be blocked by *daf-16* RNAi (Fig. 5a), suggesting that Gi/o may act upstream of DAF-16 to regulate lifespan. Further, the PTX transgene also suppressed the short-lived phenotype caused by overexpression of *gbb-1* (Fig. 5b), suggesting that Gi/o acts downstream of *gbb-1*. These data support our model that Gi/o acts downstream of GBB-1 but upstream of DAF-16.

The GABA_B_ receptor is known to pass signals from Gi/o heterotrimeric G proteins to PLCβ (refs 10, 37). The worm genome encodes one PLCβ homologue, EGL-8 (refs 41, 42). Similar to *gbb-1* mutant and PTX-transgenic worms, *egl-8* mutant worms were long-lived (Fig. 5c), consistent with the model that *egl-8* acts in the pathway. Importantly, the long-lived phenotype associated with *egl-8* mutant worms was suppressed by *daf-16* (Fig. 5c), suggesting that *egl-8* acts upstream of *daf-16*. Furthermore, loss of *egl-8* suppressed the short-lived phenotype caused by overexpression of *gbb-1* (Fig. 5d), suggesting that *egl-8* acts downstream of *gbb-1*. A prior study also showed that EGL-8 regulates lifespan in a DAF-16-dependent manner^43^. These results together support the model that G protein-PLCβ signalling transduces longevity signals from GBB-1 to DAF-16.

### DKF-2/PKD acts upstream of DAF-16 to regulate lifespan

DAF-16 is best known to be regulated by kinases^1^. We next set out to identify kinases that act downstream of PLCβ but upstream of DAF-16. Protein kinase C (PKC), a group of kinases activated by DAG and/or Ca^2+^, is a primary downstream target of PLCβ. The worm genome encodes four PKC homologues: *tpa-1, pkc-1, pkc-2* and *pkc-3*. However, none of these genes, when mutated or knocked down, gave rise to a long-lived phenotype as did *egl-8* and *gbb-1* (Supplementary Fig. 3a–d). These data suggest that PKC is unlikely to mediate the effect of EGL-8/PLCβ in longevity signalling.

**Figure 6:**
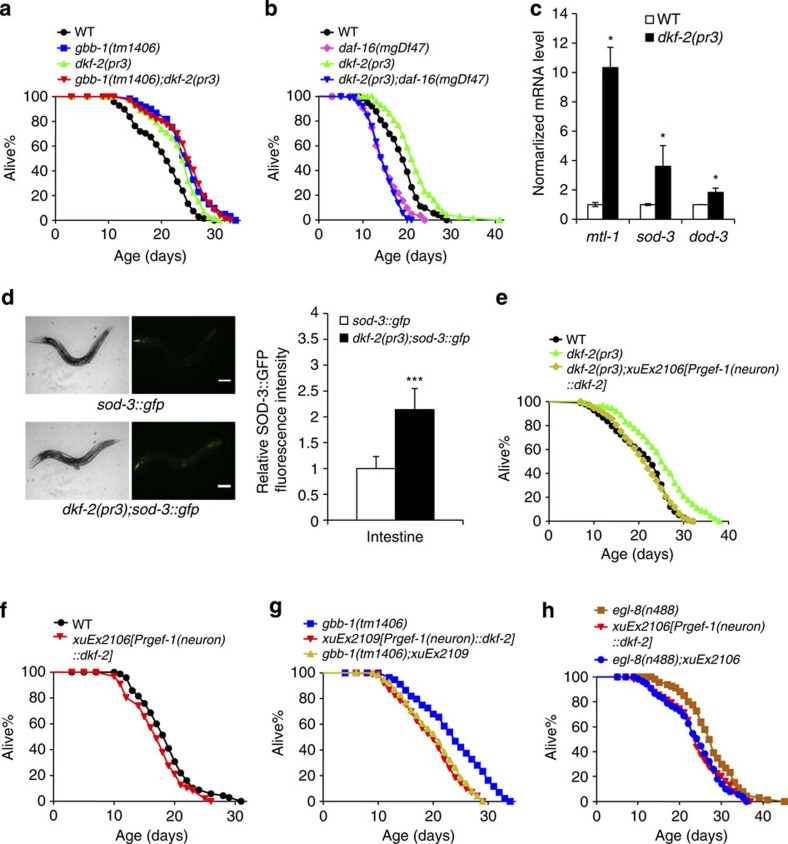
The PKD homologue DKF-2 acts downstream of G protein-PLCβ signalling but upstream of DAF-16 to regulate lifespan. (**a**) *dkf-2* acts in the *gbb-1* pathway to regulate lifespan. *dkf-2* mutant worms were long-lived (log-rank test, *P*<0.001). *dkf-2; gbb-1* double mutant showed a similar lifespan to single mutants (log-rank test, *P*=0.919), suggesting that they are in the same pathway (*n*=61–72 for different genotypes). (**b**) Loss of *daf-16* fully suppresses the long-lived phenotype of *dkf-2* mutant worms (log-rank test, *P*=0.114, *n*=72–82 for different genotypes). (**c**) qPCR analysis of DAF-16 target genes. qPCR reactions were run in triplicates for each genotype. Each experiment was repeated three times. Error bars, s.e.m. *P<0.05 (analysis of variance (ANOVA) with Bonferroni test). (**d**) Quantification of SOD-3::GFP fluorescence intensity. SOD-3::GFP is encoded by the transgene *muIs84* (ref. 46). The left panels show representative images (selected from 10 similar images). Image quantification was performed as previously described^56^. *n*≥15. Error bars, s.e.m. **P<0.001 (*t*-test). Scale bar, 100 μm. (**e**) Overexpression of *dkf-2* in neurons fully suppresses the long-lived phenotype of *dkf-2* mutant worms. *Prgef-1* is a neuron-specific promoter^62^ (log-rank test, *P*=0.143, *n*=77–98 for different genotypes). (**f**) Overexpression of *dkf-2* modestly shortens lifespan (log-rank test, *P*=0.005, *n*=58–69 for different genotypes). (**g**) Overexpression of *dkf-2* fully suppresses the long-lived phenotype of *gbb-1* mutant worms (log-rank test, *P*=0.462, *n*=44–72 for different genotypes). (**h**) Overexpression of *dkf-2* fully suppresses the long-lived phenotype of *egl-8* mutant worms (log-rank test, *P*=0.967, *n*=50–98 for different genotypes). All lifespan assays were carried out at 20 °C and were repeated at least twice. FUDR was included in assays involving *egl-8* mutant worms, which show a defect in egg laying. Please see Supplementary Table 1 for detailed statistical analysis of lifespan data.

In addition to PKC, DAG can also activate another type of kinases, protein kinase D (PKD), in both a PKC-dependent and -independent manner^44^. Two PKD homologues are found in *C. elegans*: *dkf-1* and *dkf-2* (ref. 45). We thus considered a potential role of these two PKD genes. *dkf-1* mutant worms were short-lived (Supplementary Fig. 3e–f), suggesting that it is probably not involved. By contrast, worms lacking *dkf-2* were long-lived (Fig. 6a), a phenotype similar to *gbb-1* and *egl-8* mutants. A previous study also showed that loss of *dkf-2* extends lifespan and does so by regulating DAF-16 (ref. 45). Indeed, we found that the long-lived phenotype of *dkf-2* worms was completely suppressed by loss of *daf-16* (Fig. 6b), consistent with the notion that *dkf-2* acts upstream of *daf-16* to regulate lifespan.

To obtain further evidence that *dkf-2* regulates *daf-16*, we examined whether loss of *dkf-2* can promote *daf-16* function. We found that the mRNA levels of DAF-16 target genes, such as *mtl-1, sod-3* and *dod-3*, were upregulated in *dkf-2* mutant background (Fig. 6c). We also assayed the protein level of SOD-3::GFP fusion, a commonly used reporter for DAF-16 activity, and found that it was upregulated in *dkf-2* worms (Fig. 6d). Thus, *dkf-2* appears to regulate *daf-16* function, providing additional evidence supporting that DKF-2 acts upstream of DAF-16 to control lifespan.

### DKF-2 acts downstream of EGL-8 to regulate lifespan

We then asked whether *dkf-2* functions in the *gbb-1* pathway to regulate lifespan. *dkf-2;gbb-1* double mutant exhibited a lifespan similar to single mutants (Fig. 6a), suggesting that they act in the same pathway. To gather additional evidence, we first rescued *dkf-2* long-lived phenotype by expressing wild-type *dkf-2* cDNA as a transgene in neurons in *dkf-2* mutant worms (Fig. 6e). In addition, this *dkf-2* transgene modestly shortened lifespan in wild-type background (Fig. 6f and Supplementary Table 1). Importantly, this transgene suppressed the long-lived phenotype of *gbb-1*, as well as *egl-8* mutant worms (Fig. 6g–h), providing additional evidence supporting that DKF-2 acts downstream of GBB-1 and EGL-8/PLCβ to regulate lifespan.

### Neuron-to-intestine signalling transmits longevity signals

**Figure 7:**
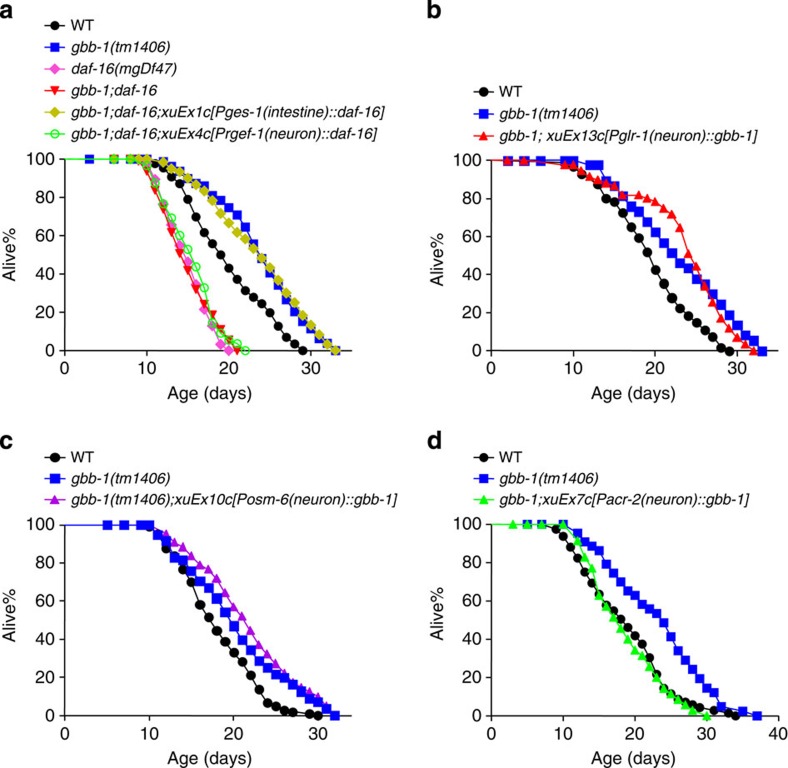
GBB-1 functions in motor neurons to signal DAF-16 in the intestine to regulate lifespan. (**a**) Transgenic expression of *daf-16* in the intestine (log-rank test, *P*=0.892 compared with *gbb-1* mutant worms), but not in neurons (log-rank test, *P*<0.001 compared with *gbb-1* mutant worms), suppresses the short-lived phenotype of *gbb-1;daf-16* double mutant worms. The *rgef-1* and *ges-1* promoters were used to drive *daf-16* cDNA expression in neurons and the intestine, respectively (*n*=55–93 for different genotypes). (**b**) Transgenic expression of *gbb-1* in subsets of interneurons fails to suppress the long-lived phenotype of *gbb-1* mutant worms. The *glr-1* promoter was used to drive *gbb-1* cDNA expression in interneurons (log-rank test, *P*<0.001, *n*=37–60 for different genotypes). (**c**) Transgenic expression of *gbb-1* in sensory neurons fails to suppress the long-lived phenotype of *gbb-1* mutant worms. The *osm-6* promoter was used to drive *gbb-1* cDNA expression in sensory neurons (log-rank test, *P*<0.001, *n*=41–103 for different genotypes). (**d**) Transgenic expression of *gbb-1* in ventral cord motor neurons fully suppresses the long-lived phenotype of *gbb-1* mutant worms (log-rank test, *P*=0.799, *n*=35–70 for different genotypes). The *acr-2* promoter was used to drive *gbb-1* cDNA expression in ventral cord motor neurons. All lifespan assays were carried out at 20 °C and were repeated at least twice. Please see Supplementary Table 1 for detailed statistical analysis of lifespan data.

Apparently, the aforementioned genetic pathway, which transmits longevity signals from GBB-1 to DAF-16, does not inform where the pathway operates. Our data showed that GBB-1 acts in neurons to regulate lifespan (Fig. 3a), yet DAF-16 is best known to function in the intestine^46^. This raises the question whether GBB-1 signals DAF-16 through neuron-to-intestine signalling. However, DAF-16 also functions in neurons^46^, which confounds our data interpretation. To clarify this issue, we interrogated the site of action of DAF-16 in the GBB-1 pathway. We found that transgenic expression of *daf-16* cDNA in the intestine but not in neurons rescued the lifespan phenotype of *gbb-1;daf-16* double mutant (Fig. 7a), indicating that DAF-16 acts in the intestine in the GBB-1 pathway.

GBB-1 is broadly expressed in the nervous system^34^. We further asked in which groups of neurons GBB-1 regulates lifespan. Transgenic expression of *gbb-1* cDNA in sensory neurons under the *osm-6* promoter did not rescue *gbb-1* mutant phenotype (Fig. 7c), nor did expression of *gbb-1* in subsets of interneurons using the *glr-1* promoter (Fig. 7b). By contrast, restoring *gbb-1* in ventral cord motor neurons through the *acr-2* promoter fully rescued the *gbb-1* longevity phenotype (Fig. 7d). Thus, GBB-1 can act in ventral cord motor neurons to regulate lifespan. This suggests a motor neuron-to-intestine signalling axis that transmits longevity signals from GBB-1 to DAF-16.

### Rat GABA_B_ receptor can functionally substitute for GBB-1

**Figure 8:**
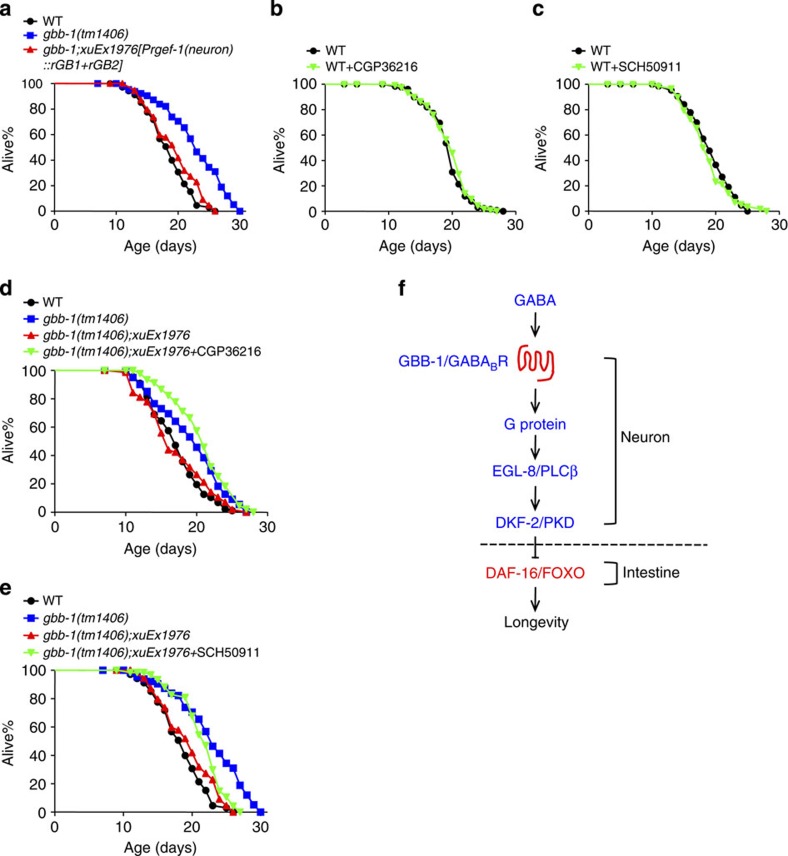
Mammalian GABA_B_ receptor can functionally substitute for worm GBB-1 in lifespan regulation. (**a**) Transgenic expression of rat GABA_B_ receptors (rGB1 and rGB2) fully suppresses the long-lived phenotype of *gbb-1* mutant worms (log-rank test, *P*=0.11, *n*=49–66 for different genotypes). (**b**,**c**) Mammalian GABA_B_ receptor antagonist CGP36216 (**b**) and SCH50911 (**c**) do not have a notable effect on lifespan in wild-type worms (log-rank test, *P*=0.389 and *P*=0.527, respectively. *n*=57–127 for the different treatments). Drug concentration: 1 μM. (**d**,**e**) Mammalian GABA_B_ receptor antagonist CGP36216 (**d**) and SCH50911 (**e**) extend the lifespan of transgenic worms expressing rat GABA_B_ receptor (log-rank test, *P*<0.001 in the each case, *n*=44–87 for the different case). Drug concentration: 1 μM antagonist. (**f**) Schematic model illustrating a genetic pathway through which GABA regulates longevity. The putative site-of-action of each gene (neuron versus intestine) is denoted. All lifespan assays were carried out at 20 °C and were repeated at least twice with the exception of **b**,**c**. Please see Supplementary Table 1 for detailed statistical analysis of lifespan data.

We expressed the rat GABA_B_ receptor, GB1/GB2 (refs 47, 48, 49), as a transgene in *gbb-1* mutant background using a neuron-specific promoter, and found that the transgene rescued the long-lived phenotype of *gbb-1* mutant worms (Fig. 8a). This suggests that mammalian GABA_B_ receptor can functionally substitute for worm GBB-1 in regulating lifespan.

Unlike worm GBB-1, the mammalian GABA_B_ receptor has been extensively characterized pharmacologically^15^,^48^,^50^,^51^. We thus took this advantage by testing some known antagonists of the GABA_B_ receptor such as CGP36216 (ref. 52) and SCH50911 (ref. 53). These chemicals did not have a notable effect on lifespan in wild-type animals (Fig. 8b,c). Remarkably, when these chemicals were applied to transgenic worms expressing the rat GABA_B_ receptor GB1/GB2, they extended lifespan and did so as efficiently as *gbb-1* mutation (Fig. 8d,e). These data present further evidence supporting that the mammalian GABA_B_ receptor can functionally substitute for worm GBB-1 in lifespan regulation, raising the possibility that it may play a similar role in mammals.

## Discussion

As the primary inhibitory neurotransmitter, GABA is best known to regulate the function and development of the brain and it plays a key role in controlling animal behaviour^11^,^12^,^13^,^14^. In the current study, we identified a new role of GABA signalling in aging. Interestingly, the effect of GABA on longevity is mediated by GABA_B_ rather than GABA_A_ receptor. As a GPCR, GABA_B_ receptor generally meditates the slow, long-lasting actions of GABA, which is consistent with its role in regulating longevity. A previous study reported that knocking down *Drosophila* GABA_B_ receptor in insulin-producing cells shortens lifespan^54^, an effect opposite to that observed in *C. elegans*. However, unlike extended lifespan, it is difficult to interpret a short-lived phenotype. As such, it remains unclear whether GABA and GABA_B_ receptor modulate aging in this organism. On the other hand, mammalian GABA_B_ receptor can functionally substitute for its worm homologue in lifespan control; furthermore, antagonists of mammalian GABA_B_ receptor can extend the lifespan of transgenic worms expressing this receptor, raising the possibility that GABA and GABA_B_ receptor may play a role in aging in other organisms. It will be interesting to test this hypothesis in future studies.

Recent studies have uncovered an increasingly important role of the nervous system in longevity^1^,^3^,^4^,^7^,^8^. Neurons regulate lifespan in a cell-nonautonomous manner presumably through neurotransmission^1^,^3^,^4^,^7^,^8^. Aside from neuroendocrine signalling, neurotransmitters are also believed to play a key role in this process. For example, endoplasmic reticulum (ER) stress in neurons affects lifespan through neurotransmitter rather than neuroendocrine signalling^55^. The identity of this neurotransmitter(s), however, remains elusive. By characterizing mutant strains defective in each of the major neurotransmitters in *C. elegans*, we found that GABA signalling plays an important role in regulating lifespan. To the best of our knowledge, this represents the first comprehensive analysis examining the role of all neurotransmitters in lifespan control in any organism.

Our results uncovered a genetic pathway that transmits longevity signals from GABA via G protein-PLCβ signalling to the FOXO transcription factor DAF-16, which is a key regulator of lifespan (Fig. 8f). Specifically, this genetic pathway consists of GBB-1, G protein, EGL-8/PLCβ, DKF-2/PKD and DAF-16/FOXO (Fig. 8f). It is interesting that PKD rather than PKC transmits signals from PLCβ in our case, although PKC plays an important role in temperature-dependent lifespan regulation in *C. elegans*^56^. One remaining question concerns how DKF-2 regulates DAF-16. The simplest model would be that DKF-2 phosphorylates DAF-16. However, we did not detect such phosphorylation in *in vitro* kinase assays (J.G., X.Z.S.X. and J.L., unpublished data). This suggests that DKF-2 likely regulates DAF-16 indirectly. Consistent with this model, we found that GBB-1 acts in motor neurons while DAF-16 functions in the intestine. Expression of DKF-2 in neurons is also sufficient to rescue *dkf-2* lifespan phenotype. Other components in the GBB-1 genetic pathway are also best known to act in the nervous system^41^,^42^. This reveals a motor neuron-to-intestine signalling axis that transmits longevity signals from GBB-1 to DAF-16. Recent studies have pointed to a critical role of such neuron-to-intestine signalling in lifespan control^55^,^57^. However, exactly how longevity signals are transmitted between neurons and intestinal cells remains a difficult question to address. Future efforts are needed to unravel the underlying mechanisms.

Despite the observation that loss of GABA signalling seems to elicit the most pronounced effect on lifespan among all the major neurotransmitters, this does not necessarily indicate that other neurotransmitters do not have a role in lifespan control. Previous studies have demonstrated that worms experience a decline in dopamine and serotonin levels with age^58^. Octopamine has also been reported to play a key role in mediating CREB-regulated transcription coactivator (CRTC)-dependent lifespan regulation in neurons^57^. These neurotransmitters all bind to multiple types of receptors, including both GPCRs and ion channels. It is possible that their receptors have opposing effects on longevity, with some promoting lifespan and others inhibiting it, which may account for the modest effect resulting from a complete loss of these neurotransmitters. It is also possible that these neurotransmitters may play a more prominent role in controlling lifespan under certain specific physiological conditions. Our studies will encourage others to investigate how the nervous system regulates lifespan through neurotransmitter signalling, an interesting but poorly understood question in the biology of aging.

## Methods

### Genetics and molecular biology

Wild-type: N2. TQ2181:*unc-13(e51).* TQ4927: *tbh-1(n3247)* × 8 outcrossed. TQ4935: *cat-2(e1112)* × 8 outcrossed. TQ4933: *eat-4(ky5)* × 8 outcrossed. TQ4929: *tdc-1(n3419)* × 8 outcrossed. TQ4931: *tph-1(mg280)* × 8 outcrossed. *unc-17(e245)* × 4 outcrossed. TQ6057: *unc-25(e156)* × 4 outcrossed. TQ4912: *gbb-1(tm1406)* × 8 outcrossed. TQ4911: *gbb-2(tm1165)* × 8 outcrossed. TQ6054: *unc-49(e407)* × 4 outcrossed. TQ5117: *xuEx1611[Pges-1::gbb-1::SL2::mCherry].* TQ5119: *xuEx1613[Pmyo-3::gbb-1::SL2::mCherry].* TQ5123: *xuEx1617[Prgef-1::gbb-1::SL2::mCherry].* TQ5842: *xuEx1964[PH20::ptx+Prgef-1::DsRed]. xuEx2117[Prgef-1::gbb-1::SL2::YFP].* TQ4914: *egl-8(n488)* × 4 outcrossed. TQ4077: *daf-16(mgDf47)* × 4 outcrossed. TQ5450: *dkf-2(pr3)* × 6 outcrossed. *xuEx2106[Prgef-1::dkf-2::SL2::mCherry], xuEx2109[Prgef-1::dkf-2::SL2::YFP]*, TQ5940: *xuEx1976[Prgef-1::rGB1::SL2::mCherry+Prgef-1::rGB2::SL2::YFP].* TQ5492: *goa-1(sa734)* × 2 outcrossed. TQ4915: *tpa-1(k530)* × 4 outcrossed. TQ5576: *pkc-1(nj3)* × 4 outcrossed. TQ5008: *dkf-1(ok2695)* × 4 outcrossed. TQ5447: *dkf-1(pr2)* × 6 outcrossed. The *pkc-3* RNA interference (RNAi) clone was generated in the laboratory, while other RNAi clones were from the Ahringer library and were confirmed by sequencing. All mutants in the GBB-1 pathway are null alleles, including *gbb-1, egl-8, dkf-2* and *daf-16*. These mutants have been outcrossed to our N2 strain for four to eight times before lifespan assay.

Microinjections were performed using standard protocols. Each plasmid DNA listed above in the transgenic line was injected at a concentration of 50 ng μl^−1^. For those experiments involving transgenes, three independent transgenic lines were tested for lifespan to confirm the results. For simplicity and clarity, only the data from one transgenic line were shown.

### Lifespan assay

Lifespan studies were performed on 60-mm nematode growth medium (NGM) plates at 20 °C as previously described^56^,^59^,^60^. For each lifespan assay, 70–110 worms were included and transferred every other day to fresh NGM plates with 14 worms per plate. The first day of adulthood was considered day 1. Survival was scored every 1–2 days, and worms were censored if they crawled off the plate, hatched inside or lost the vulva integrity during reproduction. 5-Fluoro-2′-deoxyuridine was included in assays involving *unc-13, unc-17* and *egl-8* mutant worms, which show egg-laying defects. For RNAi experiments, NGM plates included carbenicillin (25 μg ml^−l^) and isopropyl-β-D-thiogalactoside (1 mM). HT115 bacteria with vector or RNAi plasmid were seeded on RNAi plates 2 days before experiment. Worms were fed RNAi bacteria, beginning at the egg stage.

For GABA_B_ receptor antagonist experiments, 1 μM CGP36216 or SCH50911 (Tocris) was first spread out on NGM plates to let it diffuse for 1 day. L4 hermaphrodites were then transferred to these plates to assay lifespan. Worms were transferred to fresh drug plates every 2–3 days until day 10, after which animals remained on the same drug plates until death.

Graphpad Prism 5 (GraphPad Software Inc.) and IBM SPSS Statistics 19 (IBM Inc.) were used to analyse lifespan data. *P* values were calculated with the log-rank (Kaplan–Meier) method.

### Overexpression of rat GABA_B_ receptor in *C. elegans*

cDNA encoding the rat GABA_B_ receptor subunits, GB1 or GB2, was driven by the neuron-specific promoter *rgef-1*. The two plasmids were co-injected (50 ng μl^−1^ each) into *gbb-1* mutant worms to generate the transgenic animals overexpressing rat GABA_B_ receptor.

### qRT–PCR and microscopy

Total RNA was isolated from ∼200 Day-3 adult worms using TRI Reagent (Life Technologies). Quantitative PCR (qPCR) experiments were performed with CYBR Green (Life Technologies) according to the protocol provided by the manufacturer to analyse the amount of mRNA of *daf-16* target genes. qPCR data were analysed with the ΔΔ*C*_t_ method using *act-1* (actin) as an internal reference for normalization. Primer sequences used for qPCR are (all 5′–3′) listed as below. *act-1:* 5′-CCAGGAATTGCTGATCGTATGCAGAA-3′, 5′-TGGAGAGGGAAGCGAGGATAGA-3′. *mtl-1*: 5′-TGCAGTCTCCCTTACATCCA-3′, 5′-TGCAGTGGAGACAAGTGTTG-3′. *sod-3*: 5′-TATTAAGCGCGACTTCGGTTCCCT-3′, 5′-CGTGCTCCCAAACGTCAATTCCAA-3′. *dod-3*: 5′-AAAAAGCCATGTTCCCGAAT-3′, 5′-GCTGCGAAAAGCAAGAAAAT-3′.

Quantification of SOD-3::GFP fluorescence intensity was performed on an Olympus BX51 upright microscope as described previously^56^. Images were acquired with a Roper CoolSnap charge couple device camera controlled with MetaMorph (Molecular Devices Inc.) and analysed with ImageJ (NIH). SOD-3::GFP is encoded by the transgene *muIs84* (ref. 46).

## Additional information

**How to cite this article:** Chun, L. *et al*. Metabotropic GABA signalling modulates longevity in *C. elegans*. *Nat. Commun.* 6:8828 doi: 10.1038/ncomms9828 (2015).

## Supplementary Material

Supplementary Information Supplementary Figures 1-3 and Supplementary Table 1.
