# Supplementary material for: Metabotropic GABA signalling modulates longevity in C. elegans
Source: Nat Commun. 2015 Nov 5;6:8828. doi: 10.1038/ncomms9828 (PMC4667614; doi:10.1038/ncomms9828)
Supplement: Supplementary Information — Supplementary Figures 1-3 and Supplementary Table 1. [file ncomms9828-s1.pdf]

Supplementary Figure 1

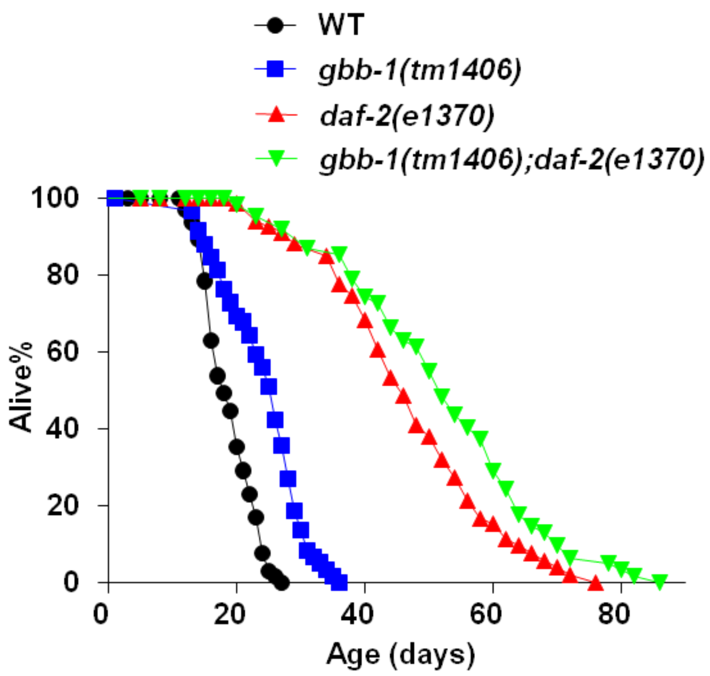

Supplementary Figure 1. Insulin/IGF Signaling pathway acts in parallel to GBB-1 pathway to modulate longevity. *gbb-1* mutation further extended the lifespan of *daf-2* mutant (log rank test, $p<0.02$, n=59-65 for different genotypes). Lifespan studies were performed on 60 mm NGM plates at 20°C and included at least three independent experiments. For each lifespan assay, 100 worms were included and transferred every other day to fresh NGM plates with 14 worms per plate. The first day of adulthood was considered day 1. Survival was scored every 1-2 days, and worms were censored if they crawled off the plate, hatched inside, or lost the vulva integrity during reproduction.

Supplementary Figure 2

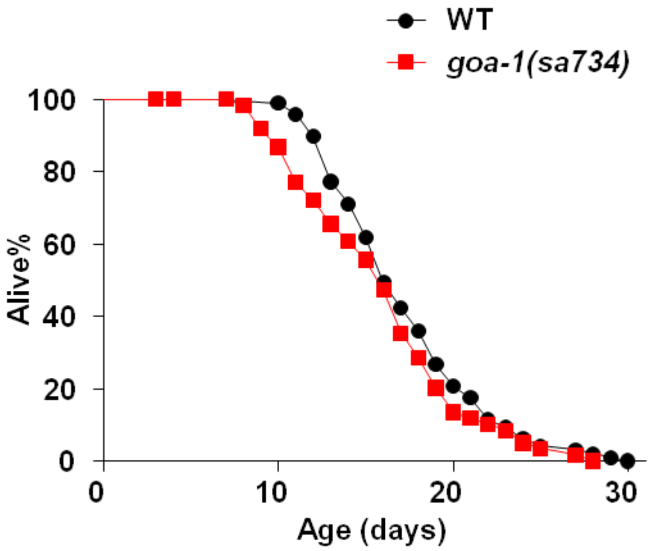

Supplementary Figure 2. Loss of the Go gene *goa-1* does not cause a long-lived phenotype. *goa-1* encodes the sole worm homolog of Go protein. (log rank test, *p*=0.18, n=60-97 for different genotypes). Lifespan studies were performed on 60 mm NGM plates at 20°C and included at least three independent experiments. For each lifespan assay, 100 worms were included and transferred every other day to fresh NGM plates with 14 worms per plate. The first day of adulthood was considered day 1. Survival was scored every 1-2 days, and worms were censored if they crawled off the plate, hatched inside, or lost the vulva integrity during reproduction.

Supplementary Figure 3

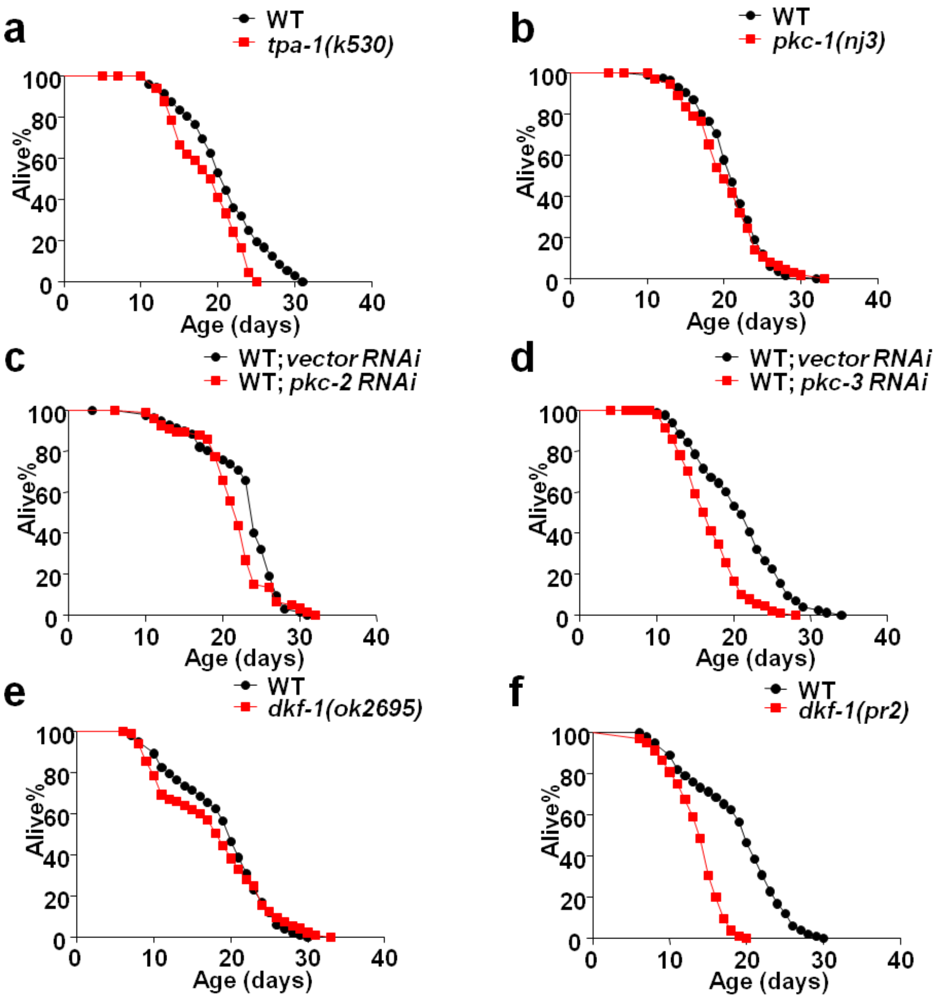

Supplementary Figure 3. Loss of worm PKC genes or the other PKD gene *dkf-1* does not give rise to a long-lived phenotype. (a-d) The worm genome encodes four PKC homologs: *tpa-1, pkc-1, pkc-2,* and *pkc-3*. Mutations or RNAi of these PKC genes either shortened lifespan or had no notable effect on lifespan (log rank test, p=0.001, p=0.577, p=0.045 and p<0.001, respectively. n=60-90 for the different genotypes). *pkc-2* null was shown to be short-lived at 20°C in a previous study (Xiao et al 2013). *pkc-3* null is lethal. **(e-f)** *dkf-2* mutant worms are not long-lived (log rank test, p=0.804 and p<0.001, respectively. n=97-105 for different genotypes). Lifespan studies were performed on 60 mm NGM plates at 20°C and included at least three independent experiments. For each lifespan assay, 100 worms were included and transferred every other day to fresh NGM plates with 14 worms per plate. The first day of adulthood was considered day 1. Survival was scored every 1-2 days, and worms were censored if they crawled off the plate, hatched inside, or lost the vulva integrity during reproduction. For RNAi experiments, NGM plates included carbenicillin (25 µg/ml) and IPTG (1 mM). HT115 bacteria with vector or RNAi plasmid were seeded on RNAi plates 2 days before experiment. The control or mutant worms were fed bacteria with vector or RNAi plasmid from egg stage.

Supplementary Table 1

Strain	Temp (°C)	Mean Lifespan ±SEM	Median Lifespan	75% Lifespan	n (assayed/total)	*P* value against control	Figure
**WT**	20	22.9±0.6	23	20	74/80	-	1a
unc-13(e51)	20	27.4±0.8	28	24	82/100	<0.001	1a
**WT**	20	22.6±0.5	22	20	91/100	-	exp.2
unc-13(e51)	20	26.1±0.9	26	22	71/100	<0.001	exp.2
**WT**	20	20.6±0.4	21	18	76/80	-	exp.3
unc-13(e51)	20	24.8±0.7	26	19	91/100	<0.001	exp.3
**WT**	20	20.4±0.7	20	15	78/100	-	1b
tbh-1(n3247)	20	20.1±0.7	20	15	79/100	0.755	1b
**WT**	20	19.8±0.5	20	16	72/100	-	exp.2
tbh-1(n3247)	20	19.8±0.6	20	17	55/100	0.843	exp.2
**WT**	20	19.3±0.4	20	18	71/100	-	exp.3
tbh-1(n3247)	20	20.0±0.4	20	18	61/100	0.325	exp.3
**WT**	20	20.4±0.7	20	15	78/100	-	1c
cat-2(e1112)	20	20.6±0.7	20	15	89/100	0.811	1c
**WT**	20	19.1±0.6	19	16	51/100	-	exp.2
cat-2(e1112)	20	18.1±0.6	18	15	48/100	0.124	exp.2
**WT**	20	17.9±0.6	18	13	68/100	-	exp.3
cat-2(e1112)	20	16.8±0.6	17	12	53/100	0.157	exp.3
**WT**	20	19.4±0.5	19	16	57/100	-	1d
eat-4(ky5)	20	19.3±0.5	19	17	33/99	0.371	1d
**WT**	20	21.1±0.7	21	16	80/100	-	exp.2
eat-4(ky5)	20	22.4±0.8	23	18	62/100	0.403	exp.2
**WT**	20	18.8±0.5	19	14	83/100	-	exp.3
eat-4(ky5)	20	19.3±0.6	19	16	51/150	0.559	exp.3
**WT**	20	20.4±0.7	20	15	78/100	-	1e
tdc-1(n3419)	20	18.8±0.7	18	15	71/100	0.046	1e
**WT**	20	17.6±0.5	17	14	62/100	-	exp.2
tdc-1(n3419)	20	17.5±0.6	17	14	48/100	0.841	exp.2

**WT**	20	19.3±0.4	20	18	71/100	-	exp.3
tdc-1(n3419)	20	17.6±0.4	18	15	71/100	0.006	exp.3
**WT**	20	20.4±0.7	20	15	78/100	-	1f
tph-1(mg280)	20	18.6±0.7	18	14	66/100	0.069	1f
**WT**	20	19.3±0.6	20	15	71/100	-	exp.2
tph-1(mg280)	20	16.7±0.9	16	11	50/99	0.07	exp.2
**WT**	20	16.6±0.6	16	13	65/100	-	exp.3
tph-1(mg280)	20	14.4±0.6	13	11	44/100	0.009	exp.3
**WT**	20	17.8±0.5	18	14	105/110	-	1g
unc-17(e245)	20	17.7±0.5	18	13	114/116	0.912	1g
**WT**	20	18.7±0.4	18	16	100/108	-	exp.2
unc-17(e245)	20	18.9±0.6	18	14	113/118	0.147	exp.2
**WT**	20	19.2±0.7	18	14	97/100	-	exp.3
unc-17(e245)	20	18.4±0.6	17	14	93/100	0.154	exp.3
**WT**	20	20.0±0.5	20	17	85/100	-	1h
unc-25(e156)	20	24.8±0.8	25	21	57/100	<0.001	1h
**WT**	20	18.2±0.6	19	15	67/95	-	exp.2
unc-25(e156)	20	20.9±0.7	21	17	40/100	0.001	exp.2
**WT**	20	19.1±0.5	19	16	64/100	-	exp.3
unc-25(e156)	20	25.0±0.7	25	21	77/100	<0.001	exp.3
**WT**	20	19.4±0.6	19	16	64/100	-	2a
gbb-1(tm1406)	20	24.4±0.8	24	19	51/100	<0.001	2a
gbb-2(tm1165)	20	20.0±0.6	20	16	67/100	0.518	2a
gbb-1(tm1406);gbb-2(tm1165)	20	24.9±0.8	24	21	44/100	<0.001	2a
**WT**	20	20.5±0.6	21	17	80/100	-	exp.2
gbb-1(tm1406)	20	24.6±0.8	23	21	57/100	<0.001	exp.2
gbb-2(tm1165)	20	21.2±0.5	21	18	80/100	0.491	exp.2
gbb-1(tm1406);gbb-2(tm1165)	20	24.7±0.7	24	21	61/100	<0.001	exp.2
**WT**	20	20.8±0.6	21	16	73/100	-	exp.3
gbb-1(tm1406)	20	25.6±0.6	25	22	71/100	<0.001	exp.3
gbb-2(tm1165)	20	21.6±0.7	22	18	61/100	0.401	exp.3
gbb-1(tm1406);gbb-2(tm1165)	20	25.1±0.7	26	22	67/100	<0.001	exp.3

**WT**	20	18.2±0.6	19	15	67/95	-	2b
unc-49(e407)	20	18.8±0.6	19	15	76/100	0.353	2b
**WT**	20	20.0±0.5	20	17	85/100	-	exp.2
unc-49(e407)	20	19.5±0.8	20	14	63/100	0.164	exp.2
**WT**	20	20.2±0.6	21	16	81/100	-	exp.3
unc-49(e407)	20	20.4±0.7	21	16	65/100	0.652	exp.3
**WT**	20	21.7±0.5	22	19	63/100	-	3a
gbb-1(tm1406)	20	26.7±0.6	27	24	59/100	<0.001	3a
gbb-1(tm1406);xuEX1611	20	26.5±0.7	26	24	47/100	<0.001	3a
gbb-1(tm1406);xuEX1613	20	25.3±0.8	25	20	52/100	<0.001	3a
gbb-1(tm1406);xuEX1617	20	20.3±0.7	20	17	54/100	0.198	3a
**WT**	20	20.4±0.5	20	17	64/100	-	exp.2
gbb-1(tm1406)	20	22.9±0.8	23	17	52/100	0.002	exp.2
gbb-1(tm1406);xuEX1611	20	22.0±0.9	22	17	35/75	0.019	exp.2
gbb-1(tm1406);xuEX1613	20	23.5±0.8	22	19	55/100	0.001	exp.2
gbb-1(tm1406);xuEX1617	20	20.5±0.9	21	17	34/78	0.591	exp.2
**WT**	20	18.4±0.5	18	16	46/100	-	exp.3
gbb-1(tm1406)	20	25.1±0.8	25	21	49/100	<0.001	exp.3
gbb-1(tm1406);xuEX1611	20	25.5±0.7	25	23	40/100	<0.001	exp.3
gbb-1(tm1406);xuEX1613	20	24.3±0.6	25	21	57/100	<0.001	exp.3
gbb-1(tm1406);xuEX1617	20	19.0±0.6	18	16	40/88	0.295	exp.3
**WT**	20	19.2±0.5	19	17	72/100	-	3b
xuEX1617	20	16.4±0.6	16	13	53/105	0.001	3b
**WT**	20	21.1±0.5	22	19	84/100	-	exp.2
xuEX1617	20	19.5±0.6	20	16	60/75	0.136	exp.2
**WT**	20	21.1±0.5	22	19	84/100	-	exp.3
xuEX1617	20	18.4±0.4	18	16	94/123	<0.001	exp.3
**WT;*daf-16 RNAi***	20	13.9±0.3	13	12	91/105	-	4a
gbb-1(tm1406);daf-16 RNAi	20	14.3±0.3	14	12	78/105	0.502	4a
**WT;*daf-16 RNAi***	20	17.9±0.5	18	14	80/105	-	exp.2
gbb-1(tm1406);daf-16 RNAi	20	17.0±0.4	17	14	87/105	0.154	exp.2
**WT;*daf-16 RNAi***	20	17.1±0.4	17	14	77/90	-	exp.3
gbb-1(tm1406);daf-16 RNAi	20	17.0±0.6	18	13	50/80	0.641	exp.3

**WT;*pha-4 RNAi***	20	17.7±0.3	18	15	89/102	-	4b
gbb-1(tm1406);pha-4 RNAi	20	20.3±0.5	20	16	79/101	<0.001	4b
**WT;*pha-4RNAi***	20	17.0±0.4	17	15	91/101	-	exp.2
gbb-1(tm1406);pha-4RNAi	20	19.6±0.5	19	15	90/103	<0.001	exp.2
**WT;*pha-4RNAi***	20	15.5±0.4	20	14	86/100	-	exp.3
gbb-1(tm1406);pha-4RNAi	20	16.7±0.4	22	14	83/100	0.025	exp.3
**WT;*skn-1 RNAi***	20	18.2±0.3	18	16	94/99	-	4c
gbb-1(tm1406);skn-1 RNAi	20	21.3±0.5	21	18	84/100	<0.001	4c
**WT;*skn-1 RNAi***	20	18.0±0.3	17	16	90/102	-	exp.2
gbb-1(tm1406);skn-1 RNAi	20	20.6±0.4	21	18	72/101	<0.001	exp.2
**WT;*skn-1 RNAi***	20	19.8±0.3	20	18	89/100	-	exp.3
gbb-1(tm1406);skn-1 RNAi	20	21.3±0.5	22	18	78/100	0.001	exp.3
**WT;*hsf-1 RNAi***	20	12.2±0.2	12	11	85/100	-	4d
gbb-1(tm1406);hsf-1 RNAi	20	13.6±0.2	14	12	80/100	<0.001	4d
**WT;*hsf-1 RNAi***	20	11.9±0.2	12	11	86/102	-	exp.2
gbb-1(tm1406);hsf-1 RNAi	20	13.2±0.2	13	12	81/100	<0.001	exp.2
**WT;*hsf-1 RNAi***	20	13.3±0.2	13	12	92/100	-	exp.3
gbb-1(tm1406);hsf-1 RNAi	20	14.2±0.2	14	13	92/100	0.003	exp.3
**WT;*vector RNAi***	20	18.2±0.5	19	14	76/105	-	5a
xuEx1964;vector RNAi	20	21.5±0.6	20	18	68/121	<0.001	5a
**WT;*daf-16 RNAi***	20	14.3±0.3	14	12	96/105	-	5a
xuEx1964;daf-16 RNAi	20	13.8±0.3	14	12	85/119	0.046	5a
**WT;*vector RNAi***	20	19.0±0.5	19	16	72/100	-	exp.2
xuEx1964;vector RNAi	20	21.8±0.6	21	19	73/100	0.001	exp.2
**WT;*daf-16 RNAi***	20	15.2±0.4	15	12	83/100	-	exp.2
xuEx1964;daf-16 RNAi	20	14.7±0.3	15	13	87/100	0.158	exp.2
**WT**	20	20.7±0.6	21	16	82/100	-	5b
xuEx2117	20	17.7±0.6	18	15	65/100	<0.001	5b
xuEx1964	20	24.4±0.8	25	20	70/100	-	5b
xuEx1964;xuEx2117	20	23.7±0.7	25	19	88/100	0.56	5b
**WT**	20	20.7±0.8	20	16	58/105	-	exp.2
xuEx2117	20	18.2±0.8	18	13	47/80	0.08	exp.2

xuEx1964	20	24.4±0.8	24	22	48/90	-	exp.2
xuEx1964;xuEx2117	20	22.8±0.7	22	19	63/105	0.078	exp.2
**WT**	20	18.9±0.5	19	15	96/113	-	5c
egl-8(n488)	20	27.7±0.7	26	23	86/103	<0.001	5c
daf-16(mgDf47)	20	14.8±0.3	15	13	96/107	-	5c
egl-8(n488);daf-16(mgDf47)	20	15.1±0.3	15	14	80/99	0.781	5c
**WT**	20	20.9±0.5	20	17	85/108	-	exp.2
egl-8(n488)	20	22.8±0.4	23	20	96/113	0.022	exp.2
daf-16(mgDf47)	20	16.6±0.3	16	15	91/112	-	exp.2
egl-8(n488);daf-16(mgDf47)	20	16.4±0.2	16	15	100/114	0.226	exp.2
**WT**	20	22.7±0.4	22	20	115/117	-	exp.3
egl-8(n488)	20	27.1±0.7	27	22	57/106	<0.001	exp.3
daf-16(mgDf47)	20	16.4±0.3	16	15	109/113	-	exp.3
egl-8(n488);daf-16(mgDf47)	20	14.6±0.3	14	13	102/111	<0.001	exp.3
**WT**	20	21.2±0.4	21	19	100/108	-	5d
xuEx1617	20	18.1±0.4	18	15	102/146	<0.001	5d
egl-8(n488)	20	28.4±0.6	27	25	78/105	-	5d
egl-8(n488);xuEx1617	20	29.0±0.7	29	26	72/118	0.382	5d
**WT**	20	22.7±0.4	22	20	115/117	-	exp.2
xuEx1617	20	20.3±0.4	21	17	82/116	<0.001	exp.2
egl-8(n488)	20	27.1±0.7	27	22	57/106	-	exp.2
egl-8(n488);xuEx1617	20	27.3±0.6	27	24	72/119	0.726	exp.2
**WT**	20	20.4±0.6	21	16	67/105	-	6a
dkf-2(pr3)	20	23.3±0.5	24	20	72/105	<0.001	6a
gbb-1(tm1406)	20	24.8±0.6	25	23	61/105	-	6a
gbb-1(tm1406);dkf-2(pr3)	20	24.7±0.6	25	22	71/105	0.919	6a
**WT**	20	18.2±0.8	18	13	47/80	-	exp.2
dkf-2(pr3)	20	20.7±0.8	20	16	58/105	0.08	exp.2
gbb-1(tm1406)	20	24.4±0.8	24	22	48/90	-	exp.2
gbb-1(tm1406);dkf-2(pr3)	20	22.7±0.7	22	19	63/105	0.078	exp.2
**WT**	20	19.0±0.6	19	16	72/106	-	exp.3
dkf-2(pr3)	20	21.2±0.6	22	17	73/108	0.011	exp.3
gbb-1(tm1406)	20	22.1±0.6	22	19	60/98	-	exp.3
gbb-1(tm1406);dkf-2(pr3)	20	21.8±0.6	21	18	81/100	0.978	exp.3
**WT**	20	19.0±0.6	19	16	72/106	-	6b
dkf-2(pr3)	20	21.2±0.6	22	17	73/108	0.011	6b

daf-16(mgDf47)	20	15.8±0.4	16	13	82/105	-	6b
dkf-2(pr3);daf-16(mgDf47)	20	15.0±0.4	14	12	77/91	0.114	6b
**WT**	20	20.1±0.4	20	18	81/105	-	exp.2
dkf-2(pr3)	20	22.4±0.6	22	19	80/105	0.002	exp.2
daf-16(mgDf47)	20	15.8±0.3	15	14	92/105	-	exp.2
dkf-2(pr3);daf-16(mgDf47)	20	16.0±0.2	16	14	87/105	0.72	exp.2
**WT**	20	19.4±0.6	21	16	64/105	-	exp.3
dkf-2(pr3)	20	22.8±0.6	24	19	70/105	<0.001	exp.3
daf-16(mgDf47)	20	15.4±0.4	15	12	83/105	-	exp.3
dkf-2(pr3);daf-16(mgDf47)	20	16.5±0.5	17	13	83/105	0.071	exp.3
**WT**	20	21.0±0.6	23	16	98/100	-	6e
dkf-2(pr3)	20	25.2±0.7	25	20	90/100	<0.001	6e
dkf-2(pr3);xuEx2106	20	22.5±0.7	23	18	77/100	0.143	6e
**WT**	20	18.2±0.8	18	13	47/80	-	exp.2
dkf-2(pr3)	20	20.7±0.8	20	16	58/105	0.08	exp.2
dkf-2(pr3);xuEx2106	20	17.4±0.6	18	14	59/102	0.218	exp.2
**WT**	20	19.0±0.6	19	16	72/106	-	exp.3
dkf-2(pr3)	20	21.2±0.6	22	17	73/108	0.011	exp.3
dkf-2(pr3);xuEx2106	20	18.0±0.6	18	15	56/102	0.326	exp.3
**WT**	20	18.8±0.5	19	16	69/102	-	6f
xuEx2106	20	17.1±0.4	17	15	58/100	0.005	6f
**WT**	20	18.9±0.6	19	16	70/100	-	exp.2
xuEx2106	20	17.2±0.6	17	14	48/102	0.064	exp.2
**WT**	20	18.8±0.6	19	16	65/94	-	exp.3
xuEx2106	20	17.2±0.5	17	14	60/122	0.039	exp.3
gbb-1(tm1406)	20	23.8±0.9	24	19	44/78	<0.001	6g
xuEx2109	20	19.5±0.6	20	15	72/100	-	6g
gbb-1(tm1406);xuEx2109	20	20.3±0.7	21	16	50/80	0.462	6g
gbb-1(tm1406)	20	28.3±0.6	27	25	66/80	0.001	exp.2
xuEx2109	20	23.7±0.4	24	20	98/120	-	exp.2
gbb-1(tm1406);xuEx2109	20	24.0±0.8	25	21	91/100	0.386	exp.2
gbb-1(tm1406)	20	23.4±0.6	23	20	62/100	0.015	exp.3
xuEx2109	20	21.0±0.6	21	18	72/100	-	exp.3
gbb-1(tm1406);xuEx2109	20	20.1±0.7	21	16	52/100	0.492	exp.3

egl-8(n488)	20	27.6±0.8	28	24	65/100	0.002	6h
xuEx2106	20	23.9±0.7	24	20	98/100	-	6h
egl-8(n488);xuEx2106	20	23.9±0.9	25	19	50/70	0.967	6h
egl-8(n488)	20	27.1±0.7	27	22	57/106	<0.001	exp.2
xuEx2106	20	21.8±0.6	23	17	100/100	-	exp.2
egl-8(n488);xuEx2106	20	21.5±0.6	21	17	88/100	0.863	exp.2
**WT**	20	19.9±0.5	19	16	86/105	<0.001	7a
gbb-1(tm1406)	20	23.8±0.6	24	20	79/100	-	7a
daf-16(mgDf47)	20	15.0±0.3	15	13	93/105	<0.001	7a
gbb-1;daf-16	20	15.0±0.3	15	12	91/105	<0.001	7a
gbb-1;daf-16;xuEx1c	20	23.6±0.7	24	19	60/82	0.892	7a
gbb-1;daf-16;xuEx4c	20	15.5±0.4	16	13	55/89	<0.001	7a
**WT**	20	19.4±0.7	19	15	77/90	<0.001	exp.2
gbb-1(tm1406)	20	23.9±0.8	24	20	45/78	-	exp.2
daf-16(mgDf47)	20	15.6±0.3	15	14	89/99	<0.001	exp.2
gbb-1;daf-16	20	15.5±0.3	15	14	82/102	<0.001	exp.2
gbb-1;daf-16;xuEx1c	20	22.9±0.7	23	18	68/92	0.583	exp.2
gbb-1;daf-16;xuEx4c	20	14.6±0.4	14	11	72/82	<0.001	exp.2
**WT**	20	19.6±0.7	20	16	54/70	-	7b
gbb-1(tm1406)	20	23.0±1.0	23	18	37/81	0.001	7b
gbb-1(tm1406);xuEx13c	20	23.5±0.7	25	21	60/87	<0.001	7b
**WT**	20	19.4±0.7	19	15	77/90	-	exp.2
gbb-1(tm1406)	20	23.9±0.8	24	20	45/78	<0.001	exp.2
gbb-1(tm1406);xuEx13c	20	23.9±0.8	25	20	71/99	<0.001	exp.2
**WT**	20	18.2±0.4	18	15	103/150	-	7c
gbb-1(tm1406)	20	20.4±0.8	20	15	56/150	0.003	7c
gbb-1(tm1406);xuEx10c	20	21.7±0.9	22	18	41/150	<0.001	7c
**WT**	20	20.0±0.6	20	15	97/150	-	exp.2
gbb-1(tm1406)	20	22.8±1.1	22	17	39/150	0.006	exp.2
gbb-1(tm1406);xuEx10c	20	23.4±1.2	23	16	43/150	<0.001	exp.2
**WT**	20	19.6±0.7	20	16	54/70	-	exp.3
gbb-1(tm1406)	20	23.0±1.0	23	18	37/81	0.001	exp.3
gbb-1(tm1406);xuEx10c	20	23.9±0.9	25	19	54/82	<0.001	exp.3
**WT**	20	18.7±0.7	19	14	70/100	-	7d
gbb-1(tm1406)	20	23.2±1.0	24	17	42/90	<0.001	7d

gbb-1(tm1406);xuEx7c	20	18.7±0.9	18	15	35/90	0.799	7d
**WT**	20	19.5±0.6	19	15	81/150	-	exp.2
gbb-1(tm1406)	20	22.2±1.0	23	16	51/150	0.007	exp.2
gbb-1(tm1406);xuEx7c	20	19.0±0.7	18	14	61/150	0.611	exp.2
**WT**	20	20.0±0.6	20	15	97/150	-	exp.3
gbb-1(tm1406)	20	22.8±1.1	22	17	39/150	0.006	exp.3
gbb-1(tm1406);xuEx7c	20	20.6±0.8	21	15	60/150	0.266	exp.3
**WT**	20	18.4±0.4	19	16	66/92	-	8a
gbb-1(tm1406)	20	22.8±0.6	23	19	59/87	<0.001	8a
gbb-1(tm1406);xuEx1976	20	19.4±0.5	20	16	49/117	0.11	8a
**WT**	20	17.8±0.5	19	14	56/107	-	exp.2
gbb-1(tm1406)	20	20.7±0.7	21	17	47/106	<0.001	exp.2
gbb-1(tm1406);xuEx1976	20	17.8±0.7	17	14	60/108	0.486	exp.2
**WT**	20	19.4±0.5	19	16	75/105	-	exp.3
gbb-1(tm1406)	20	23.9±0.8	25	19	46/105	<0.001	exp.3
gbb-1(tm1406);xuEx1976	20	20.4±0.6	20	18	41/91	0.55	exp.3
**WT**	20	19.3±0.3	20	18	127/147	-	8b
**WT+CGP36216**	20	19.5±0.3	20	18	114/140	0.389	8b
**WT**	20	18.2±0.4	19	17	63/98	-	8c
**WT+SCH50911**	20	18.7±0.5	19	16	57/100	0.527	8c
**WT**	20	17.2±0.4	17	14	87/112	-	8d
gbb-1(tm1406)	20	19.3±0.6	20	15	56/116	0.001	8d
**gbb-1(tm1406);*xuEx1976***	20	17.0±0.6	16	14	59/84	0.705	8d
gbb-1(tm1406);xuEx1976+CGP36213	20	20.7±0.6	21	17	44/84	<0.001	8d
**WT**	20	17.8±0.5	19	14	56/107	-	exp.2
gbb-1(tm1406)	20	20.7±0.7	21	17	47/106	<0.001	exp.2
**gbb-1(tm1406);*xuEx1976***	20	17.8±0.6	17	14	60/108	0.486	exp.2
gbb-1(tm1406);xuEx1976+CGP36213	20	19.5±0.7	19	15	59/99	0.024	exp.2
**WT**	20	18.4±0.4	19	16	66/92	-	8e
gbb-1(tm1406)	20	22.8±0.6	23	19	59/87	<0.001	8e
**gbb-1(tm1406);*xuEx1976***	20	19.4±0.5	20	16	49/117	0.11	8e
gbb-1(tm1406);xuEx1976+SCH50911	20	21.5±0.5	22	20	51/111	<0.001	8e
**WT**	20	17.8±0.5	19	14	56/107	-	exp.2
gbb-1(tm1406)	20	20.7±0.7	21	17	47/106	<0.001	exp.2

**gbb-1(tm1406);*xuEx1976***	20	17.8±0.7	17	14	60/108	0.486	exp.2
gbb-1(tm1406);xuEx1976+SCH50911	20	19.4±0.5	20	17	63/110	0.063	exp.2

The Log Rank (Mantel-Cox) test was used for statistical analysis. N numbers are described as: assayed/total, i.e. the number of assayed animals/total animals included to the plates initially. The difference represents the number of animals that were censored (crawled off the plate, bagged or exploded). FUDR was included in lifespan assays involving *unc-13*, *unc-31* and *egl-8 mutant worms* to suppress internal hatching of eggs.
